# Melanoma’s New Frontier: Exploring the Latest Advances in Blood-Based Biomarkers for Melanoma

**DOI:** 10.3390/cancers16244219

**Published:** 2024-12-18

**Authors:** Ivana Prkačin, Mislav Mokos, Nikola Ferara, Mirna Šitum

**Affiliations:** 1 Department of Dermatology and Venereology, Sestre Milosrdnice University Hospital Center, 10000 Zagreb, Croatia; mislav.mokos@kbcsm.hr (M.M.); nikola.ferara@kbcsm.hr (N.F.); mirna.situm@kbcsm.hr (M.Š.); 2 School of Medicine, University of Split, 21000 Split, Croatia; 3 School of Dental Medicine, University of Zagreb, 10000 Zagreb, Croatia; 4 Croatian Academy of Sciences and Arts, 10000 Zagreb, Croatia

**Keywords:** melanoma, biomarkers, S100B, LDH, liquid biopsy, circulating tumor cells, cell-free DNA, circulating tumor DNA, cell-free RNA, tumor-educated platelets

## Abstract

Melanoma is one of the most malignant cancers, and the global incidence of cutaneous melanoma is increasing. While melanomas are highly prone to metastasize if diagnosed late, early detection and treatment significantly reduce the risk of mortality. Identifying patients at higher risk of metastasis, who might benefit from early adjuvant therapies, is particularly important, especially with the advent of new melanoma treatments. Therefore, there is a pressing need to develop additional prognostic biomarkers for melanoma to improve early stratification of patients and accurately identify high-risk subgroups, ultimately enabling more effective personalized treatments. Recent advances in melanoma therapy, including targeted treatments and immunotherapy, have underscored the importance of biomarkers in determining prognosis and predicting treatment response. The clinical application of these markers holds the potential for significant advancements in melanoma management. Various tumor-derived genetic, proteomic, and cellular components are continuously released into the bloodstream of cancer patients. These molecules, including circulating tumor DNA and RNA, proteins, tumor cells, and immune cells, are emerging as practical and precise liquid biomarkers for cancer. In the current era of effective molecular-targeted therapies and immunotherapies, there is an urgent need to integrate these circulating biomarkers into clinical practice to facilitate personalized treatment. This review highlights recent discoveries in circulating melanoma biomarkers, explores the challenges and potentials of emerging technologies for liquid biomarker discovery, and discusses future directions in melanoma biomarker research.

## Simple Summary

This review provides a comprehensive overview of advancements in melanoma biomarkers, emphasizing the potential of serologic biomarkers for early diagnosis, prognosis, and personalized treatment of melanoma. Notable markers include S100B and lactate dehydrogenase (LDH), already partially integrated into clinical practice, and new candidates like melanoma-inhibiting activity (MIA), osteopontin, and tumor-associated antigen 90 immune complex (TA90-IC), which offer predictive capabilities for treatment outcomes. New biomarkers, including genetic, proteomic, and cellular markers, are emerging as crucial tools. These biomarkers, such as circulating tumor DNA (ctDNA) and RNA, can offer real-time insights into tumor dynamics, enabling non-invasive monitoring through liquid biopsies. Additionally, tumor-educated platelets and circulating immune cells show promise in understanding melanoma’s aggressive behavior. Lastly, the review discusses the integration of these biomarkers into clinical protocols and the need for continued research to establish these tools’ accuracy, improving patient-specific treatment strategies.

## 1. Introduction

Melanoma, a highly aggressive and potentially lethal form of skin cancer, continues to represent a significant global health challenge [1]. The incidence of melanoma has been steadily increasing, with an estimated 200,340 new cases expected to be diagnosed in the U.S. in 2024, including 99,700 non-invasive [in situ] and 100,640 invasive cases [2]. Despite the availability of effective treatments, including surgical resection in the early stages, the prognosis for advanced melanoma remains poor [3]. Accurate and early diagnosis is essential for improving patient outcomes, but the complexity of melanoma and its ability to mimic benign melanocytic lesions, such as nevi, pose substantial diagnostic difficulties [4].

Traditionally, melanoma diagnosis heavily relies on histopathologic examination [5]. Even though this approach is usually effective, there are cases where the distinction between melanoma and benign lesions is not clear-cut, even for experienced pathologists [4]. Tumors with overlapping histopathologic features, such as certain types of nevi or rare melanoma subtypes like amelanotic and desmoplastic melanoma, can be particularly challenging to diagnose [6]. In such cases, immunohistochemistry (IHC) assists in diagnosis. Commonly used markers in IHC are sensitive for detecting melanoma cells but lack specificity, as they may also be expressed in benign melanocytic lesions [7]. As a result, their utility in differentiating between malignant and benign melanocytic proliferations is limited. This diagnostic ambiguity emphasizes the need for more reliable molecular biomarkers that can aid in early and accurate melanoma diagnoses.

In addition to diagnostic challenges, melanoma also presents a considerable prognostic dilemma. Current clinical and histopathologic parameters, including Breslow thickness, ulceration, mitotic rate, and sentinel lymph node status, are well-established predictors of patient outcomes [8]. However, these factors fail to account for the behavior of aggressive melanoma subtypes, which may rapidly progress despite being thin, non-mitotically active, or non-ulcerated skin lesions [9]. Therefore, all of these limitations point to a critical need for new prognostic biomarkers, which can effectively predict disease behavior and guide clinical decision-making.

Over the past decade, the management of advanced melanoma cases has been revolutionized by new molecularly targeted therapies and immune checkpoint inhibitors [10]. The discovery of oncogenic drivers, particularly BRAF mutations, has enabled the development of targeted therapies that inhibit crucial proteins in the mitogen-activated protein kinase [MAPK] pathway, such as BRAF and MEK inhibitors [11,12]. These treatments have shown considerable success in extending survival in patients with metastatic melanoma, particularly those with BRAF V600 mutations. For example, combination therapies involving BRAF and MEK inhibitors, such as dabrafenib and trametinib, have significantly improved overall survival (OS), with a median survival of 25 months and a 5-year survival rate of 28% [13]. Nevertheless, the efficacy of these therapies is limited by the emergence of resistance, with many patients developing acquired resistance within 9–12 months of treatment initiation [14,15].

In parallel, immunotherapy has emerged as a powerful therapeutic option for melanoma. Immune checkpoint inhibitors, particularly those targeting the CTLA-4 and PD-1 pathways, have demonstrated durable responses and extended survival in a subset of patients [16,17]. However, despite these advances, many patients do not achieve long-term disease control, with resistance [either primary or acquired] remaining a significant barrier to successful treatment [18]. Thus, it is evident that understanding the underlying resistance mechanisms and identifying serologic melanoma biomarkers, which correctly predict therapeutic responses, is essential for improving patient outcomes and optimizing treatment strategies.

Moreover, the development of biomarkers is critical not only for improving outcomes in advanced melanoma but also for shaping the future of adjuvant therapies in earlier stages of the disease [19]. Recent clinical trials have begun to explore the use of biomarkers as secondary endpoints, helping to determine which melanoma patients are most likely to benefit from adjuvant-targeted therapies or immunotherapies [20]. Furthermore, biomarkers are already guiding treatment sequencing and identifying the optimal timing for therapy [21].

Melanoma biomarkers can be categorized into three main types: diagnostic, prognostic, and predictive. They can also be classified based on their tissue- or blood-based origin.

Diagnostic biomarkers strengthen early detection of melanoma, providing a more reliable distinction between melanoma and benign lesions than current histopathologic and IHC methods [22]. Prognostic biomarkers may estimate melanoma progression and natural clinical outcomes without targeted or systemic therapies. These biomarkers are particularly valuable at the time of initial diagnosis of melanoma since they provide estimates of the tumor’s aggressiveness, the potential for metastasis, and the overall prognosis [7,22]. Predictive biomarkers may help assess the patient’s response to a specific treatment, such as BRAF or immune checkpoint inhibitors. By identifying potential responders and non-responders, these biomarkers are particularly valuable in the context of personalized medicine [23].

**Figure 1:**
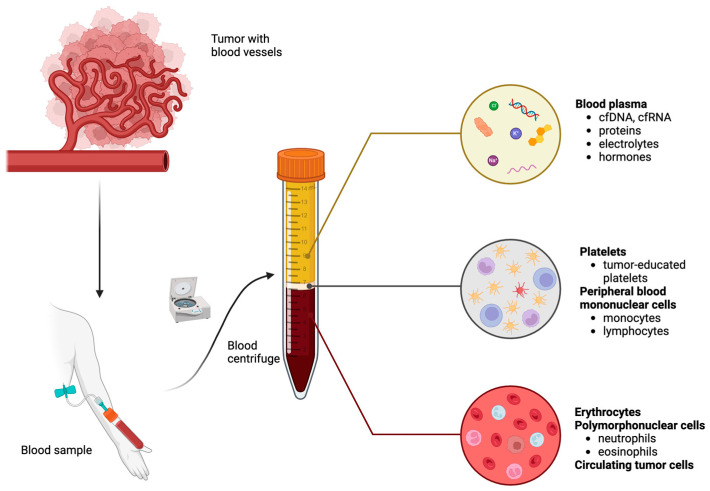
Peripheral blood centrifugation by density gradient with components of each layer. Abbreviations: cfDNA: cell-free DNA; cfRNA: cell-free RNA. Created in BioRender. Mokos, M. (2024) https://BioRender.com/x74l463.

This review will provide an overview of current and emerging blood-based biomarkers in melanoma while focusing on their potential applications in diagnosis, prognosis, and personalized therapy. Blood-based biomarkers will include serologic biomarkers and those identified in biological liquid samples, termed liquid biopsy biomarkers (Figure 1). We will also discuss the current limitations and future directions of biomarker research, highlighting the importance of integrating new technologies and biomarker discoveries into clinical practice.

## 2. Serologic Biomarkers

### 2.1. Lactate Dehydrogenase

Lactate dehydrogenase (LDH) is an enzyme found across nearly all tissues in the body. It is produced by two genes: LDH-A, which forms the M (muscle) subunit, and LDH-B, responsible for the H (heart) subunit. These subunits combine in various ways to create five distinct isoenzymes. The primary function of LDH is to accelerate the conversion of pyruvate into lactate, which generates ATP [24]. LDH is a widely distributed enzyme found in large amounts in the liver, kidneys, heart muscle, skeletal muscles, and red blood cells [25]. Recent research highlights LDH’s significant role in tumor development. LDH is key in the Warburg effect, a process where cancer cells shift from aerobic metabolism to primarily anaerobic glycolysis, converting glucose into lactate. This metabolic shift is commonly observed in malignant cells, and it is important in their ability to promote tumor growth [26].

Scientific evidence clearly demonstrates the significant prognostic value of LDH in melanoma, which is one of the primary reasons it is the only serologic biomarker incorporated into the American Joint Committee on Cancer (AJCC) staging system [27]. A retrospective study analyzed 334 stage IV cutaneous melanoma cases, all with documented serum LDH levels, and it revealed that elevated LDH levels correlated with an increased likelihood of death. Cox multivariable survival analysis confirmed that as LDH levels rose, so did the mortality risk [28].

Moreover, it has been shown that LDH is an independent predictive factor in patients treated with targeted and immunotherapy. High levels of LDH in blood plasma correlated with a poor prognosis in melanoma patients who received trametinib and dabrafenib therapy [29]. Furthermore, research has shown that a positive response to immunotherapy in melanoma patients is linked to a significant decrease in LDH serum levels [30].

### 2.2. S100B

S100B, a protein with an uncommon solubility in a fully saturated ammonium sulfate solution, is a member of the S100 protein family, which now consists of over 20 calcium-binding proteins, mainly homodimers with structural similarities and varying amino acid homology across different tissues [31]. S100B, an acidic homodimer with a monomer weight of 9–14 kDa, was long considered brain-specific but has since been detected in other cell types [32].

The serum concentration of S100 appears to be associated with tumor size and malignancy [33]. This link between S100 proteins and tumor progression may be due to their role in tumorigenesis. Specifically, S100 proteins inhibit calcium-dependent phosphorylation of p53 by protein kinase C, which could disrupt p53-mediated apoptosis regulation, leading to unchecked tumor growth [34]. Elevated S100 levels have been observed in various tumors like prostate, breast, gastric, bladder, lung, thyroid, kidney, and oral cancers, with the highest levels found in malignant melanoma [35].

S100B’s role as a potential prognostic biomarker in melanoma stems from its involvement in disease progression. High levels of S100B in the serum are linked to poorer survival rates and increased relapse risk [33]. Some studies suggest that S100B may surpass LDH in predicting OS in metastatic melanoma [36]. Though recommended in some national and international guidelines for relapse detection, its use remains limited outside these countries due to conflicting data regarding its prognostic value [37]. Acland et al. have proved that S100 serum levels are not helpful in predicting the presence of micrometastases in primary cutaneous melanoma [38]. On the other hand, a study by Jury et al. indicates that increasing serum levels of S100 protein serves as a precise and sensitive marker that is clinically significant for monitoring tumor progression in melanoma patients, often appearing before other signs of disease recurrence [39]. Furthermore, several studies demonstrated that serum S100 was a more reliable prognostic indicator than urinary metabolites, and that the relative risk of mortality increased by five times when serum S100 levels exceeded the threshold of 0.6 μg/L [40,41].

The systematic review and meta-analysis of Janka et al. revealed that serum S100B had a significantly better capacity to detect disease recurrence than serum LDH. The prognostic value of serum S100B, which was reflected in its adjusted hazard ratio (HR) of 1.78, was shown to be independent but not significantly better than that of serum LDH, which had an HR of 1.60 [33]. Also, S100B may serve as a reliable baseline marker for OS in melanoma patients undergoing anti-PD-1 therapy. An increase in S100B levels during the initial weeks of treatment could provide valuable insights to guide therapeutic decisions [42]. Moreover, S100B shows potential as a valuable tool for monitoring response to anti-CTLA-4 therapy, particularly after the first two doses of ipilimumab [43,44].

### 2.3. Melanoma-Inhibiting Activity

Melanoma-inhibiting activity (MIA) is a soluble protein with a molecular weight of 12 kDa that is secreted by melanoma cells and is considered a melanoma-specific serologic biomarker [45]. Early research has demonstrated that MIA is significantly present in various melanoma cell lines and tissues. Additionally, it has been shown that MIA disrupts cell-to-cell interactions between melanoma cells and the extracellular matrix, facilitating the migration and metastasis of these cancer cells [46]. From a clinical point of view, elevated serum levels of MIA at the time of diagnosis are associated with a higher risk of recurrence. Similar to S100B, research indicates that MIA exhibits greater sensitivity, specificity, and accuracy in identifying melanoma metastasis compared to other tumor markers [47]. Furthermore, Bolovan et al. concluded in their study that serum levels of MIA, in conjunction with the expression of several melanoma tissue markers, could enhance the process of stratifying patients at high risk for tumor progression [48]. Also, Li et al. found that serum levels of MIA were significantly elevated in patients with melanoma compared to age-matched healthy donors. Moreover, MIA levels were sigificantly higher in patients with melanoma stages III–IV compared to stages I–II. A cut-off value of >914.7 pg/mL for MIA predicted disease progression with high specificity [86.4%] and sensitivity [95.5%] [49].

Sanmamed et al. have reported that MIA and S100B decrease significantly after the onset of treatment with BRAF inhibitors, while lower concentrations after the beginning of treatment are associated with longer PFS [50]. Furthermore, Uslu et al. have shown that S100 and MIA are highly sensitive tumor markers for monitoring melanoma patients receiving therapeutic vaccination immunotherapy with dendritic cells. MIA demonstrated slightly higher sensitivity than S100 in detecting disease progression and appears to be particularly valuable for monitoring metastatic melanoma patients undergoing immunotherapy [51].

### 2.4. Vascular Endothelial Growth Factor

Vascular endothelial growth factor (VEGF) is a glycoprotein composed of two identical subunits with a combined molecular weight of around 45 kDa [52]. It is a key growth factor that plays a crucial role in promoting new blood vessel formation. It stimulates endothelial cells to multiply, helps prevent cell death, enhances blood vessel permeability, and supports cell movement. These actions make VEGF essential in managing both normal and abnormal processes of blood vessel growth [53].

Serum levels of VEGF are known to be elevated in various types of cancer [54,55,56].

Wang et al. observed elevated serum levels of VEGF-A in patients with both primary and metastatic melanoma. Moreover, VEGF-A levels showed a positive association with Breslow thickness, indicating a link between VEGF-A concentration and tumor depth [57]. Moreover, Lugowska et al. reported significantly higher serum levels of VEGF in patients with stage I–III melanoma [58].

Tas et al. have found that serum VEGF are significantly higher in melanoma patients than in healthy individuals. In this study, VEGF levels were influenced by Breslow thickness and mitotic rate but were not associated with disease stage [59].

A possible explanation for these findings could lie in the fact that the VEGF/VEGFR-signaling axis, encompassing factors like VEGF-A, PlGF, VEGF-B, VEGF-C, and VEGF-D, along with receptors VEGFR-1, VEGFR-2, and VEGFR-3, plays a vital role in melanoma by driving angiogenesis. This pathway initiates complex cellular-signaling cascades involving the MAPK/ERK, PI3K/AKT, PKC, PLC-γ, and FAK pathways. Through these interconnected signals, the VEGF/VEGFR axis significantly impacts melanoma cell behaviors, including growth, proliferation, migration, metastasis, survival, and the development of resistance to treatments [60].

Furthermore, elevated baseline serum VEGF levels are linked to reduced OS and lower response rates to ipilimumab therapy, indicating that serum VEGF could be a predictive biomarker for treatment outcomes with immune checkpoint inhibitors [61].

### 2.5. Osteopontin

Osteopontin (OPN) is a highly acidic glycophosphoprotein with an extensive phosphorylation and high aspartic acid content. Its function is known in many health conditions, including cardiovascular diseases, cancer, diabetes, and kidney stone formation. It also contributes to key biological processes such as inflammation, biomineralization, cell survival, and wound repair [62]. Lately, OPN has been recognized as a biomarker for melanoma.

Maier et al. have investigated the potential of OPN as a serologic biomarker for detecting metastatic melanoma. The predictive accuracy, evaluated using the area under the receiver operating characteristic (AUC) curve, was 0.85 for OPN, 0.89 for S100, and 0.69 for LDH. Notably, combining OPN with S100 yielded a higher AUC of 0.97. This combined biomarker approach achieved a sensitivity of 95.5% and a specificity of 85.9% at the optimal cut-off value [63]. Similarly, Várvölgyi et al. evaluated the diagnostic value of LDH, S100B, and osteopontin for detecting melanoma metastases. Osteopontin and S100B levels significantly predict metastasis independently. Combining these biomarkers with tumor localization and AJCC pT category improved diagnostic accuracy, achieving high discrimination [64]. Another study revealed that higher OPN levels were associated with advanced AJCC stages at sampling. Untreated stage IV patients had significantly elevated levels compared to treated stage I–III patients. While higher OPN levels showed a trend toward increased mortality risk, it was not statistically significant [65].

Moreover, Prasmickaite et al. have assessed serum OPN as a potential biomarker for identifying high-risk melanoma patients with poor prognosis or those who might benefit from adjuvant interferon (IFN)-α therapy. While patients with high OPN levels showed slightly improved survival with IFN-α treatment, the difference was not statistically significant. The study concluded that OPN levels or genotypes do not reliably predict prognosis or response to IFN-α in melanoma patients [66].

### 2.6. Interleukin 8

Interleukin (IL)-8 is a chemokine crucial for promoting inflammation and the formation of new blood vessels. Its expression is notably elevated in tumor cell lines, tumor tissues, and in the peripheral blood of cancer patients [67]. Evidence across various tumor types suggests that elevated baseline levels of IL-8 are associated with poorer clinical outcomes [68,69].

It is long known that IL-8 levels are elevated in melanoma patients [70]. Furthermore, in melanoma patients, serum IL8 levels were found to be linked with tumor size and stage, survival rates, and measurable treatment responses, including responses to BRAF inhibitors and immune-targeting monoclonal antibodies [71].

In melanocytes, IL-8 production has only been observed following stimulation by cytokines such as TNF or IL-1β, which initiate pro-inflammatory responses [72]. In melanoma cell lines, baseline IL-8 mRNA expression levels have been found to correlate with metastatic potential, while non-tumorigenic, non-metastatic cell lines show no detectable IL-8 mRNA expression [73]. This evidence strongly suggests that pro-inflammatory cytokines, like IL-8, could play a significant role in driving the development of metastases in areas affected by inflammation.

Also, research further indicates that IL-8 plays a direct role in fostering resistance to both chemotherapy and targeted therapies [74,75]. More recent clinical studies have linked IL-8-driven myeloid cell infiltration in tumors with resistance to immune checkpoint inhibitors (ICIs), suggesting that peripheral IL-8 levels can predict response to ICI therapy [76]. It is known that targeting IL-8 or its receptors can enhance immune cell-mediated tumor destruction, with combination treatments blocking the IL-8/IL-8R axis alongside ICIs demonstrating increased anti-tumor effectiveness. Based on these findings and IL-8’s role as a prognostic marker, multiple clinical trials are now underway to investigate the benefits of incorporating IL-8 targeting into immune-based therapies [77,78].

### 2.7. Tyrosinase

Tyrosinase, a copper-dependent oxidase enzyme, is essential for melanin biosynthesis. It facilitates two critical reactions in the melanin-production pathway: the hydroxylation of tyrosine to L-DOPA and the oxidation of L-DOPA to dopaquinone [79]. Studies on the role of tyrosinase in melanoma have evolved significantly over the years as detection techniques have advanced.

Early studies, like the one of Sonesson et al., focused on developing a method for measuring tyrosinase activity in blood serum. Healthy subjects exhibited tyrosinase values ranging from 0.1 to 1.0 nkatal/L, while melanoma patients showed significantly higher levels ranging from 1.1 to 10.6 nkatal/L. These findings indicated elevated serum tyrosinase activity in melanoma metastasis patients compared to healthy controls [80].

Later technological advances allowed scientists to explore newer techniques for tyrosinase detection in blood serum. For example, the study of Stevens et al. evaluated an RT-PCR assay’s sensitivity for detecting tyrosinase mRNA in peripheral blood and its correlation with melanoma status. The authors suggested that RT-PCR detection of tyrosinase mRNA is feasible and may serve as a stage-related marker for melanoma, warranting further research [81].

Furthermore, reduced-sensitivity PCR has been proven to be more clinically relevant for detecting tyrosinase mRNA in melanoma patients [82].

Another study showed that combining S-100 and RT-PCR for tyrosinase mRNA can be of clinical use, since positive RT-PCR was most informative in patients with S-100 levels < 0.15 µg/L. S-100 demonstrated higher predictive value overall, but RT-PCR for tyrosinase mRNA added useful prognostic data in select cases [83].

Recent studies investigated the role of antibodies against melanocyte differentiation antigens, including tyrosinase, as predictive markers for immune checkpoint inhibitor [ICI] therapy in melanoma patients. Higher baseline levels of antibodies against melanocyte differentiation antigens, such as tyrosinase, were associated with better responses to ICI therapy and improved survival outcomes. This suggests that these antibodies could serve as predictive biomarkers for melanoma patients undergoing ICI treatment [84].

### 2.8. Other Melanoma Non-Specific Serologic Biomarkers

Beyond the biomarkers already covered, a wide array of other serum biomarkers shows considerable promise.

One such marker, the tumor-associated antigen 90 immune complex (TA90-IC), has demonstrated potential usefulness in melanoma detection and monitoring. Chung et al. found that postoperative TA90-IC levels are strongly predictive of outcomes in patients with thick primary melanomas. Patients who tested negative for TA90-IC had significantly better disease-free survival (DFS) and OS rates than those who tested positive [85]. Furthermore, the presence of TA90-IC in blood serum, along with a lack of anti-TA90 IgM antibodies, is linked to distant metastasis in low- or intermediate-risk melanoma patients [86]. In addition, Kelley et al. reported that the TA90-IC assay is a powerful tool for predicting survival and identifying subclinical disease following melanoma surgery, aiding in the selection of patients for adjuvant therapy. Namely, the assay showed the ability to detect disease recurrence an average of 19 months earlier than standard clinical and radiographic evaluations, potentially enabling earlier and more effective therapeutic interventions [87].

TA90-IC holds the distinction of being the first serum marker demonstrated to predict survival outcomes in melanoma patients undergoing adjuvant immunotherapy following complete resection of distant metastases. In the study of Hsueh et al., stage IV melanoma patients were treated with postoperative PMCV immunotherapy, and their pre-vaccine TA90-IC levels showed a strong correlation with both OS and DFS, underscoring its significance as a prognostic tool in this context [88]. Furthermore, Tsioulias et al. reported that a polyvalent allogeneic whole-cell vaccine triggers the production of TA90-IC in melanoma patients with subclinical disease. Patients who undergo seroconversion for TA90-IC exhibit significantly better DFS and OS compared to those who remain TA90-IC positive. This makes TA90-IC a valuable prognostic indicator and a potential surrogate marker for evaluating the clinical effectiveness of polyvalent allogeneic whole-cell vaccine therapy [89]. Also, the study by Faries et al. suggested that TA90-IC and MIA are complementary in tracking recurrence in stage III melanoma patients who underwent adjuvant vaccine immunotherapy [90].

YKL-40, also known as Chitinase-3-Like Protein 1 (CHI3L1), is a well-preserved glycoprotein that non-enzymatically binds to heparin and chitin. It serves as a valuable biomarker for the early detection, prognosis, and monitoring of various inflammatory diseases, including those affecting the cardiovascular, gastrointestinal, endocrine, immune, musculoskeletal, neurological, respiratory, urinary, and infectious systems [91]. Ismail et al. examined whether high plasma YKL-40 levels correlate with increased mortality in melanoma patients. Their results revealed that measured high plasma YKL-40 levels were significantly associated with higher mortality risk [92]. Several studies have proven that high YKL-40 levels in blood plasma correlate with poorer OS in melanoma patients [93,94,95,96]. Furthermore, Krogh et al. have hypothesized that high YKL-40 levels in blood serum could be used as an indicator of adjuvant interferon therapy [96].

The human matrix metalloproteinase (MMP) family consists of 25 enzymes divided into five main categories: collagenases, gelatinases, stromelysins, membrane-type MMPs (MT-MMPs], and others. MMPs are critical in virtually all biological processes involving matrix breakdown and remodeling [97]. They are essential in embryonic development and tissue repair and are also involved in various diseases, including chronic inflammatory conditions and cancer [98]. The previously mentioned study comparing blood samples from stage I–III melanoma patients and healthy controls found that MMP-2 levels were similar between the two groups. However, serum MMP-9 levels were notably elevated in melanoma patients compared to controls. Despite this increase, there was no correlation between MMP-9 levels and DFS or OS [58]. However, a study by Nikkola et al. has linked high serum MMP-9 levels to significantly poorer OS and metastases formation. In contrast, serum levels of MMP-1 and MMP-13 showed no correlation with OS. Elevated MMP-1 levels were associated with faster disease progression after therapy, but MMP-9 and MMP-13 levels did not correlate with time to progression [TTP]. Multivariate analysis identified MMP-9 as an independent prognostic factor for OS and MMP-1 for TTP, irrespective of age and gender [99]. Another study reported that high serum MMP-9 levels correlate with extensive metastases and reduced survival rates in melanoma patients, while elevated MMP-1 levels are associated with faster disease progression post-therapy initiation [100]. Also, higher MMP-2 serum concentrations were associated with advanced tumor stages and the presence of metastases [101]. Further research is needed to explore the significance of these data.

Vitamin D is a fat-soluble steroid hormone, which plays a key role in regulating calcium and phosphate metabolism [102]. Numerous epidemiological studies indicate that insufficient vitamin D levels may elevate the risk of certain cancers. Vitamin D interacts with the vitamin D receptor (VDR), activating the transcription of various genes that inhibit MAPK signaling, promote apoptosis, and regulate the cell cycle. This provides vitamin D with anti-proliferative and pro-apoptotic effects across many cell types. Additionally, it suppresses adaptive immune responses while reportedly enhancing innate immunity [103]. Patients with metastatic melanoma exhibited poorer prognoses when diagnosed with vitamin D deficiency and inadequate replenishment during treatment [104]. Also, low vitamin D levels were found to be an independent predictor of an increased risk of death from melanoma compared to those with merely suboptimal levels [105]. Furthermore, vitamin D levels below 9.25 ng/mL were associated with histologic ulceration and lower OS [106]. However, vitamin D supplementation does not improve relapse-free survival, melanoma-related mortality, or OS [107].

Contemporary insights into the clinical relevance of the above-described serologic biomarkers for melanoma are summarized in Table 1.

## 3. Liquid Biopsy Markers

Liquid biopsy (LB) involves real-time examination of tumor cells or their byproducts that are released into the bloodstream or other bodily fluids by primary or metastatic tumors. In contrast to traditional tissue biopsies, which are invasive and challenging to perform repeatedly, LB can be collected at multiple time points during treatment, providing real-time insights into the biology of the tumor [25]. Therefore, LB facilitates the creation of novel approaches for the early detection of initial cancers or recurrence of disease, assessment of treatment effectiveness, and identification of therapeutic targets and resistance mechanisms. This allows for therapy to be tailored to meet the unique needs of each patient [108]. Emerging research has identified various proteins, cell-free DNA (cfDNA), circulating tumor DNA (ctDNA), cell-free RNA (cfRNA), circulating tumor cells (CTCs), tumor-educated platelets, and circulating immune cells as potential biomarkers for melanoma [109,110].

### 3.1. Circulating Tumor Cells

Circulating tumor cells (CTCs) are cells that shed from the primary tumor and then enter and travel through the bloodstream [111]. Analyzing CTCs is a useful method for understanding the metastatic nature of cancer and tracking disease progression. Also, it holds promise for personalized cancer treatment. Insights from single-cell genomics and transcriptomics have revealed the complexity of CTCs, showing that they are heterogeneous at various levels and that only a small portion can initiate metastasis [112]. Additionally, CTCs appear to boost their metastatic potential through strategies like forming homotypic clusters and interacting heterotypically with immune and stromal cells [113]. Clinically, counting CTCs and conducting molecular analyses could offer new ways to monitor cancer progression and guide personalized treatment choices. However, using CTCs for early cancer detection remains challenging compared to other tumor-related biomarkers, such as circulating tumor DNA [114].

Research so far indicates that CTCs serve as an effective biomarker for assessing disease status. Additionally, certain studies have demonstrated that measuring CTC levels before and during melanoma treatment can be used for evaluating prognosis and treatment response in melanoma patients [115,116].

In a recent study by Li et al., a high initial CTC count was associated with substantial local invasion, lymph node metastasis, and distant spread. Furthermore, elevated baseline CTC levels indicated poorer overall survival and served as an independent prognostic marker. Variations in CTC counts were linked to both progression-free survival [PFS] and disease-specific survival (DSS) [117]. Moreover, Lucci et al. have demonstrated that the presence of at least one baseline CTC was significantly linked to a reduced recurrence-free survival (RFS) at both 6 months and 54 months [118]. Similarly, a study by Hall et al. showed that CTC-positive patients at baseline faced a higher risk of worse PFS within 180 days compared to those who were CTC-negative. Also, 51% of CTC-positive patients experienced a relapse, while only 15% of those without CTCs relapsed [119].

While quantifying CTCs demonstrates significant promise, the function of melanoma circulating tumor cells (MelCTCs) in managing melanoma is still being explored. The persistence or increase of MelCTCs after treatment could be a sign of disease progression. On the other hand, a decrease or a stable zero count could speak for a positive response to therapy. Moreover, changes in CTC levels post-treatment might hint at resistance emergence, prompting early adjustments in treatment. Even though assessing these fluctuations with low MelCTC counts can be challenging, such evaluations could provide valuable prognostic insights. Additionally, analyzing MelCTCs for tumor molecular evolution before and during treatment could help identify resistance markers [120].

### 3.2. Cell-Free Nucleic Acids

In addition to tumor cells, circulating nucleic acids originating from tumors have also demonstrated prognostic value. These biomarkers encompass cell-free DNA (cfDNA), circulating tumor DNA (ctDNA), and cell-free RNA.

#### 3.2.1. Cell-Free DNA

Cell-free DNA (cfDNA) consists of double-stranded fragments of deoxynucleic acid that are typically shorter than 200 base pairs, with a low molecular weight and low concentration [121]. This DNA is found in cell-free portions of blood, such as plasma and serum, as well as in various other bodily fluids [122]. Rather than being bound to cells, cfDNA often exists in complexes with proteins or within membrane-bound structures [123]. Although its exact function and origin remain unclear, several studies suggest potential sources and mechanisms through which cfDNA is released into the bloodstream [124].

cfDNA is being explored for its clinical relevance in conditions like autoimmune diseases, stroke, myocardial infarction, and transplant rejection [125]. For instance, high cfDNA levels in systemic lupus erythematosus patients are linked to antibody levels and lupus nephritis, though its role in disease activity and prognosis remains unclear [126]. In rheumatoid arthritis, cfDNA levels in serum show inconsistent patterns, further complicating its diagnostic value [127].

The concentration of cfDNA in blood shows considerable variation, ranging from 0 to over 1000 ng/mL in cancer patients and from 0 to 100 ng/mL in healthy individuals [128].

Research by Váraljai et al. indicates that total cfDNA could serve as a melanoma biomarker independent of tumor genotype. The findings suggest that cfDNA levels could be used for assessing tumor burden and risks for disease progression and mortality [129]. Furthermore, baseline cfDNA concentration was found to correlate with pre-treatment tumor burden. Higher levels of cfDNA were significantly associated with increased mortality risk and OS, with a threshold of 89 pg/μL distinguishing two prognostic groups. Patients with cfDNA levels at or above this cutoff experienced shorter OS, a finding that remained significant when compared to lactic dehydrogenase [LDH] in multivariate analysis. Also, changes in cfDNA levels indicated treatment-related alterations in tumor burden, and the ratio of baseline cfDNA to tumor burden acted as a prognostic indicator [130].

#### 3.2.2. Circulating Tumor DNA

Circulating tumor DNA (ctDNA) is gaining attention as a highly promising biomarker for early cancer detection, as tumors release ctDNA into the bloodstream well before they become detectable through imaging or other signs of disease appear [131]. ctDNA enters the bloodstream primarily through tumor cell death, such as apoptosis or necrosis, and from circulating tumor cells. As a component of cell-free DNA (cfDNA), ctDNA represents only a small portion of cfDNA, with cfDNA also containing DNA shed by non-cancerous cells [132].

Levels of circulating tumor DNA (ctDNA) differ significantly across various cancer types. For instance, ctDNA is more frequently detected in advanced pancreatic, ovarian, colorectal, gastroesophageal, breast, and melanoma cancers than primary brain, kidney, and thyroid cancers [133]. A possible explanation for this phenomenon could lie in the uniqueness of various tumor locations, as barriers like the blood–brain barrier may restrict ctDNA release into body fluids [134].

Reduced levels of circulating ctDNA are linked to a smaller tumor burden and are often indicative of better clinical responses to therapies, particularly with BRAF inhibitors. Additionally, ctDNA has the potential to track the emergence of resistance to targeted therapies by identifying resistance mutations, and it can provide early signs of disease progression even before changes are visible through imaging [135]. Therefore, they could serve as a valuable complement to traditional imaging methods in tracking disease resistance and progression.

However, ctDNA is often undetectable in the early stages of melanoma. Namely, early research on this subject by Daniotti et al. revealed that patients with stage I or II melanoma did not have detectable levels of ctDNA in their blood serum. On the other hand, their quantitative analysis showed that melanoma patients had elevated levels of circulating free DNA compared to healthy controls, with the highest concentrations found in samples collected before surgery and in those with stage IV disease. Furthermore, researchers analyzed the relationship between circulating DNA and corresponding tumors, finding a significant correlation specifically in those with stage IV melanoma [136].

Newer publications also predict that research and utilization of ctDNA will enhance liquid biopsy techniques, contributing to early prediction, tracking of disease progression, and the precise adjustment of treatment strategies for melanoma patients. A systematic review with meta-analysis by Feng et al. analyzed nine articles, covering a total of 617 melanoma patients. The combined hazard ratios (HRs) indicated that patients with detectable ctDNA at baseline had a strong association with worse OS and PFS compared to those with undetectable ctDNA. A meta-analysis of the adjusted HRs further confirmed that baseline ctDNA presence correlated with poorer OS and PFS outcomes. Additionally, ctDNA levels measured during treatment were associated with reduced OS and PFS [137]. Another meta-analysis yielded similar results, as it confirmed that there was a connection between OS and both detectable ctDNA at baseline and after treatment. For PFS, baseline detectable ctDNA appeared to correlate with poorer PFS outcomes, while both elevated baseline ctDNA levels and increases in ctDNA were significantly associated with unfavorable PFS. Patients with BRAFV600 ctDNA mutations at baseline showed a significantly worse OS compared to those who were ctDNA-negative at baseline. However, there were no significant differences in PFS between the groups with detectable and undetectable BRAFV600 ctDNA mutations at baseline [138]. In addition, examining methylated ctDNA through methylation-specific PCR in metastatic melanoma has shown encouraging links, particularly the hypermethylation of the RASSF1A-promoter region, which is significantly associated with OS [139]. Also, research has indicated that hypermethylation of the estrogen receptor-α is a predictor of both PFS and OS in melanoma patients [140].

From a therapeutic point of view, the absence of detectable ctDNA, combined with a complete response on imaging after halting immunotherapy due to toxicity, is associated with a strong likelihood of sustained long-term disease control. Conversely, patients with ongoing immunotherapy-related toxicity who still have detectable ctDNA after 8–12 weeks face a considerably elevated risk of disease progression [141]. Moreover, pre-treatment ctDNA is found to be a predictor of patient outcomes in the first-line setting for immune checkpoint inhibitors (ICIs), but this reliability diminishes in the second-line ICI context, particularly among patients who have previously received BRAF/MEK inhibitors. Initial findings suggest that treatment-naïve patients with elevated ctDNA levels might experience greater benefits from combined ICI therapies [142].

Gray et al. reported that low baseline ctDNA levels [<10 copies/mL] were associated with better therapeutic response and longer PFS in patients treated with BRAF/MEK inhibitors or anti-PD1 immunotherapy. Although patients treated with immunotherapy showed less pronounced ctDNA changes, low baseline levels still predicted favorable outcomes [143]. Moreover, ctDNA detection is associated with lower RFS and worse distant metastasis-free survival (DMFS) [144].

#### 3.2.3. Cell-Free RNA

Circulating RNA sequencing is widely used in clinical research to identify cell-free RNA (cfRNA) biomarkers associated with diseases such as cancer, infections, and autoimmune conditions. When cfRNA is released into the extracellular matrix, it affects cell signaling by facilitating communication between cells. Similarly to ctDNA, cfRNA often enters circulation passively, diffusing from apoptotic, tumor, or necrotic cells and providing a valuable window into cellular activity and disease states [145].

cfRNA has been researched in various cancer-oriented studies, including lung, hepatocellular, pancreatic, breast, nasopharyngeal, ovarian, and colorectal cancer, as well as multiple myeloma [146]. Analyzing cfRNA in blood plasma or serum offers valuable diagnostic insights, supporting early cancer detection, tumor identification, monitoring, and evaluating treatment responses [146].

Diagnostic, prognostic, and predictive accuracy of cfRNA were shown in the study, which comprised 175 melanoma stage II–IV patients treated with immune checkpoint inhibitors or targeted therapy [147].

A recent study by Wang et al. discovered that RNA methylation reduces RNA’s immunogenicity, potentially helping tumors evade immune detection. In a study of 1564 melanoma patients across eight cohorts, researchers analyzed m6A RNA modification patterns of 21 gene signatures in tumor cells. They used principal component analysis to assign each patient a score, reflecting their m6A RNA modification level. Patients fell into two groups: those with high m6Ascores had immune-excluded tumors and a 52.2% 5-year survival rate, while those with low m6Ascores showed immune-inflamed tumors and a 61.1% survival rate. Notably, low m6Ascores also predicted a stronger response to anti-PD-1 and anti-CTLA4 therapies, highlighting m6Ascore’s potential for guiding personalized immunotherapy in melanoma treatment [148].

MicroRNAs (miRNAs) are small, naturally occurring RNAs that control gene expression after transcription by reducing the expression of specific messenger RNAs [mRNAs] with sequences matching the miRNA [149]. This process is similar to how both natural and synthetic small interfering RNAs (siRNAs) function. Interestingly, many miRNAs target other noncoding RNAs rather than mRNAs [150]. miRNAs primarily enter the bloodstream through active secretion rather than from tumor cell death. In circulation, they are typically encased in lipid-based exosomes or bound to stabilizing proteins like argonaute 2 (AGO2) and nucleophosmin, making them highly stable biomarkers. Although present in very low concentrations, miRNAs can still be reliably detected using qRT-PCR. The specific miRNA expression differences observed between cancer patients and healthy individuals likely stem from controlled release by tumor and immune cells, not passive release due to cell death. This miRNA exchange via blood is thought to facilitate cell-to-cell communication, though the exact mechanisms for miRNA release and uptake remain unclear [151].

miRNAs are essential regulators of cancer-related processes such as cell growth, movement, and programmed cell death. They achieve this by targeting oncogenes, acting as tumor-suppressing miRNAs, or by influencing tumor suppressor genes as oncomiRs [151]. Many miRNAs are involved in melanoma development and progression, with evidence showing that miR-221 and miR-222 specifically target the cell-cycle regulator p27 in the Me1402/R melanoma cell line [152].

One of the pioneering studies in the field of microRNAs in melanoma patients showed that miRNA expression in blood cells is a promising biomarker for melanoma detection. Namely, Leidinger et al. identified 51 miRNAs with altered expression in melanoma patients’ blood cells: 21 were downregulated and 30 were upregulated compared to healthy controls. Validation of these results showed a strong correlation in expression changes. miRNA profiles successfully distinguished melanoma patients from healthy individuals via hierarchical clustering and principal component analysis. By utilizing 16 key deregulated miRNAs, the study achieved a classification accuracy of 97.4%, with 95% specificity and 98.9% sensitivity [153].

Several studies have demonstrated the prognostic potential of miRNAs in melanoma patients. A study by Tian et al. provides strong evidence that miR-206 may play a role in the aggressive progression of melanoma. Furthermore, serum levels of miR-206 show promise as a noninvasive biomarker for predicting prognosis in melanoma patients [154]. In addition, Stark et al. evaluated a 17-miRNA panel (MELmiR-17) in melanoma tissues and serum from Australian and German biobanks. In tissue samples, MELmiR-17 accurately predicted stage, recurrence, and survival. A blood-based, seven-miRNA subset (MELmiR-7) detected melanoma with high sensitivity (93%) and specificity (≥82%), and it outperformed standard markers (LDH and S100B) for predicting OS. This minimally invasive test shows promise for monitoring disease progression and relapse, especially in early metastatic melanoma [155].

The diagnostic and prognostic relevance of miRNAs in melanoma was confirmed in a recent study, which included 60 patients with stage I–IV melanoma [156].

### 3.3. Tumor-Educated Platelets

Emerging research reveals that platelets play a role in cancer beyond their traditional functions in homeostasis and clotting. In the tumor microenvironment, platelets interact closely with cancer cells, leading to changes in their adhesion molecules, glycoproteins, nucleic acids, proteins, and receptors, a process known as “education”. These tumor-educated platelets (TEPs) then circulate through the body, actively contributing to tumor growth and spread. This unique modification of platelets positions them as promising blood-based biomarkers, potentially useful for assessing prognosis and treatment response [157].

Platelets actively support tumor cell growth, survival, spread, and metastasis by forming a bioactive shield around CTCs, protecting them from an immune attack. This protective relationship involves both direct contact and indirect signaling (“virtual education”) by which cancer cells “train” circulating platelets to assist in shielding and promoting tumor progression [157].

There is limited information available regarding tumor-educated platelets in melanoma. Experiments by Liu et al. demonstrate that tumor-educated platelets from mouse models with melanoma exhibit increased speed and distance in migration, resulting in enhanced thrombus formation. However, plasma from tumor-bearing mice appears to inhibit platelet migration. Furthermore, the RNA sequencing of tumor-educated platelets indicates a significant upregulation of numerous genes linked to cell migration and cytoskeletal structure [158]. Furthermore, research by Chen et al. investigated the role of tumor cell-induced platelet aggregation in the progression of melanoma and its impact on the tumor immune microenvironment. They concluded that TCIPA accelerates lung metastasis in melanoma patients by utilizing TEPs to recruit tumor-associated macrophages, facilitate their polarization to the M2 phenotype, and alter the suppressive tumor immune microenvironment in lung metastases [159].

### 3.4. Circulating Immune Cells

The presence of immune cells in tumor tissue indicates melanoma’s response to immunotherapy, suggesting that analyzing immune cells may replace direct tumor tissue analysis [160].

Recent studies have shown that immune profiling is a valuable tool for identifying biomarkers that can predict the clinical outcome of melanoma patients. The study by Huang et al. profiled immune responses in stage IV melanoma patients treated with pembrolizumab, focusing on exhausted CD8 T cells in the blood. Most patients showed immune activation. However, clinical outcomes were influenced by an imbalance between T-cell reinvigoration and tumor burden. The extent of Tex cell reinvigoration relative to pretreatment tumor burden was linked to treatment success, offering a potential predictor of response to PD-1 therapy [161]. Furthermore, in a study by Martens et al., elevated absolute lymphocyte counts (ALC) between 2 and 8 weeks, along with higher percentages of CD4+ and CD8+ T cells from 8–14 weeks following initial ipilimumab treatment, were linked to better survival outcomes. Although these associations fell short of significance after strict adjustments for multiple testing, they still aligned with clinical responses. Notably, 36% of patients who showed increases in all three factors experienced favorable outcomes, with survival rates of 93.3% at 12 months and 63.8% at 24 months. Among these patients, 71% achieved partial or complete responses, in contrast to only 8% of patients who had decreases in one or more of the factors. Changes in regulatory T cells and myeloid-derived suppressor cells were not related to OS [162]. Furthermore, in the study by Wistuba–Hamprecht et al., 137 late-stage melanoma patients received treatment with ipilimumab, and their blood mononuclear cells were analyzed both before and during treatment. High baseline levels of CD8 effector-memory type 1 (EM1) T-cells were linked to longer OS and better response rates, while high levels of late-stage differentiated CD8 cells were associated with shorter OS. Additionally, a decrease in CD8 cells following treatment was consistently linked to improved clinical responses. These findings suggest that baseline CD8 EM1 levels and CD8 cell changes during treatment may be key predictors of ipilimumab response [163].

Table 2 provides an overview of recent findings on the clinical relevance of different liquid biopsy markers for melanoma.

## 4. Future Directions

Currently, no reliable blood-based biomarkers exist for the early detection or prognosis of melanoma. An effective biomarker could significantly enhance early diagnosis. However, single-serum markers have generally shown inadequate sensitivity and specificity, which limits their clinical utility. As a promising alternative, biomarker panels might offer greater diagnostic precision and predictive accuracy.

Advancements in proteomic profiling may eventually enable the identification of melanoma-specific molecular signatures. This could provide new tools for assessing the stage of melanoma, improving its treatment, and predicting patient outcomes. Ideally, a comprehensive biomarker panel would facilitate individualized staging, enable closer therapeutic monitoring, support prognostic assessment, and potentially aid in early detection, especially for high-risk patients.

Moreover, the next research phase will likely focus on biomarkers that predict treatment response, particularly to immunotherapies. Such markers would empower clinicians to tailor individualized melanoma treatment. This means that every patient would receive the most effective options for them, which could potentially enhance therapeutic outcomes.

Liquid biopsies offer multiple advantages. They are non-invasive, cost-effective, and capture material shed from various metastatic sites, which can provide a more comprehensive picture of tumor heterogeneity. Their ability to be collected serially allows for continuous monitoring of therapeutic responses and detection of minimal residual disease post-treatment [164]. Additionally, liquid biopsies hold potential in early cancer screening, making them a valuable tool for both real-time treatment adjustments and early diagnosis without the need for repeated tissue biopsies [165].

The immense potential of liquid biopsy in oncology is just beginning to be tapped effectively, with significant implications for research and clinical practice. A growing body of literature now underscores the promising clinical applications of liquid biopsies, a trend likely to strengthen in the coming years as more clinical studies incorporate repeated blood sampling into their protocols. Additionally, the rapid advancements in precise, ultra-sensitive technologies seen recently are expected to enable an even wider array of uses for the tumor-derived components present in various bodily fluids. Although this review focuses on blood-based applications, other bodily fluids also show promise as sources for liquid biopsy [166].

## 5. Challenges

Despite its promise, liquid biopsy faces significant challenges, primarily due to technological limitations that hinder effective detection and analysis.

The process of isolating CTCs generally involves two main steps. Firstly, CTCs are separated from the dense background of millions of blood cells, and then, within this enriched sample, the CTCs are identified and analyzed. Enriching and detecting CTCs in patients is particularly problematic, primarily because only small blood samples are available, and the concentration of CTCs in peripheral blood is extremely low [167]. In melanoma, these challenges increase as standard CTC markers like EpCAM [commonly used in detecting CTCs in epithelial cancers such as breast and prostate] are often absent in melanoma CTCs. This is because melanocytes originate from the neural crest, unlike epithelial-derived cells, making melanoma-specific detection markers necessary [168]. Moreover, MelCTCs represent a highly diverse group of cells [169]. However, most existing methods for isolating melanoma cells from blood overlook this variability, as they typically depend on detecting only one or two specific markers to identify CTCs [170].

The presence of ctDNA and cfDNA in the bloodstream can be minimal, especially in patients with a lower tumor load, making detection challenging [171]. Due to the limited quantity of DNA fragments available, sequencing becomes both technically complex and costly. Additionally, to achieve consistent results across different labs, standardized protocols and equipment calibration are necessary to enhance reproducibility [172]. It is important to note that not all cfDNA variations are cancer-specific; many can arise from clonal hematopoiesis of indeterminate potential [CHIP], particularly in older patients, which may lead to confounding results [173]. Furthermore, ctDNA/cfDNA shedding is not uniform across primary tumors and metastases, raising questions about whether detected genetic alterations accurately reflect the full spectrum of tumor heterogeneity [174]. Treatments can also suppress ctDNA shedding, and certain anatomical sites may yield very limited ctDNA, complicating disease monitoring [175].

## 6. Conclusions

This review highlights the significant potential of serologic biomarkers in advancing melanoma diagnosis, prognosis, and treatment personalization. Established biomarkers such as S100B and LDH are already partially embedded in clinical protocols, primarily for monitoring recurrence and predicting survival. However, their limitations underscore the need for more comprehensive tools, as biomarkers like MIA, osteopontin, and TA90-IC offer new solutions for tracking disease progression and predicting therapeutic responses.

Innovations in liquid biopsy technologies, including ctDNA and cfRNA, provide minimally invasive, dynamic insights into tumor biology. These biomarkers allow real-time monitoring of tumor dynamics, resistance mechanisms, and treatment efficacy, potentially overcoming the invasiveness and limited repeatability of traditional tissue biopsies. Additionally, novel cellular components, such as tumor-educated platelets and circulating immune cells, have shown promise in elucidating melanoma’s metastatic pathways and immune-evasion strategies, offering new avenues for monitoring disease aggression.

Despite promising advances, implementing these biomarkers in routine practice faces technological and interpretative challenges, particularly regarding the standardization of ctDNA, CTCs, and cfDNA analysis.

Future research should prioritize robust validation studies and the development of comprehensive biomarker panels. Such panels could improve diagnostic precision, stage-specific monitoring, and predictive capabilities by combining molecular and biomarkers. Ultimately, as immunotherapies and targeted treatments evolve, integrating these biomarkers into clinical protocols is essential for maximizing therapeutic outcomes and personalizing treatment strategies for melanoma patients.

## Figures and Tables

**Table 1:** Clinical relevance of different serologic biomarkers for melanoma.

Biomarker	Clinical Use	Key Findings	Limitations	References
LDH	Prognostic	↑ LDH correlates with ↑ risk of death	Not specific for melanoma	[28]
	Predictive	↑ LDH correlates with ↓ PFS and ↓ OS in pts treated with targeted and immunotherapy		[29,30]
S100B	Prognostic	↑ S100B correlates with ↓ survival rates and ↑ relapse risk S100B could surpass LDH in predicting OS S100B is relevant for monitoring tumor progression	Conflicting data regarding its prognostic value Not helpful in predicting micrometastases	[33,36,37,38,39,40,41]
	Predictive	S100B may serve as a reliable baseline marker for OS in melanoma patients undergoing anti-PD-1 and anti-CTLA-4 therapy		[33,42,43,44,50]
MIA	Prognostic	Significantly higher MIA levels in melanoma patients with stages III–IV compared to stages I–II ↑ MIA is associated with ↑ risk of recurrence and tumor progression,	Low specificity in early metastatic melanoma	[47,48,49]
	Predictive	↓ MIA after the onset of BRAF inhibitors Sensitive for monitoring melanoma pts receiving vaccination immunotherapy with dendritic cells		[50,51]
VEGF	Prognostic	↑ VEGF is associated with ↓ OS VEGF levels correlate with Breslow thickness	Low specificity and sensitivity	[57,58,59,61]
	Predictive	↑ VEGF levels are associated with ↓ response rates to ipilimumab therapy		[61]
OPN	Prognostic	OPN detection may predict metastatic disease ↑ OPN is associated with ↑ mortality risk	Not specific for melanoma	[63,64,65,66]
IL-8	Prognostic	↑ IL-8 correlates with tumor size, stage, and survival rates	Not specific for melanoma	[70,71]
	Predictive	IL-8 may predict response to BRAF inhibitors and immune-targeting monoclonal antibodies IL-8 is implicated in fostering resistance to both chemotherapy and targeted therapies IL-8 can predict response to ICI therapy		[71,74,75,76,77,78]
Tyrosinase	Prognostic	Serum tyrosinase activity in metastatic melanoma pts Serum tyrosinase activity correlates with melanoma stage	Wide range of variability in different studies	[80,81,83]
	Predictive	Higher baseline levels of TRP1/TYRP1 and TRP2/TYRP2 specific antibodies were associated with better responses to ICI therapy		[84]
TA90-IC	Prognostic	Positive TA90-IC is associated with better DFS and OS Presence of TA90-IC and absence of anti-TA90 IgM antibodies are associated with distant metastasis	Not specific for melanoma	[85,86,87]
	Predictive	TA90-IC may predict survival outcomes in melanoma pts undergoing adjuvant immunotherapy TA90-IC correlates with clinical effectiveness of polyvalent allogeneic whole-cell vaccine therapy TA90-IC and MIA are complementary in tracking recurrence after adjuvant vaccine immunotherapy		[88,89,90]
YKL-40	Prognostic	Plasma YKL-40 is associated with ↑ mortality risk ↑ plasma YKL-40 correlates with ↓ OS	Not specific for melanoma Low sensitivity	[92,93,94,95,96]
	Predictive	↑ serum YKL-40 predicts response to adjuvant IFN therapy, while YKL-40 contributes to melanoma progression by stimulating the PD-1/PD-L1 axis and other checkpoint molecules		[96]
MMPs	Diagnostic	↑ MMP-9 levels in melanoma pts compared to controls	Contradicting data	[58,99,100]
	Prognostic	Some studies correlate ↑ MMP-9 serum levels with ↓ survival rates		
Vitamin D	Prognostic	↓ vitamin D correlated with poor prognosis in pts with metastatic melanoma, ↑ mortality, and histologic ulceration	Limited data	[104,105,106]

Abbreviations: ↓: decreased; ↑: increased; DFS: disease-free survival; ICI: immune checkpoint inhibitors; IFN: interferon; IL-8: Interleukin-8; LDH: lactate dehydrogenase; MIA: melanoma inhibitory activity; MMP: matrix metalloproteinase; OPN: osteopontin; OS: overall survival; PFS: progression-free survival; pts: patients; TA90-IC: tumor-associated antigen 90 immune complex; VEGF: Vascular endothelial growth factor.

**Table 2:** Clinical relevance of different liquid biopsy biomarkers for melanoma.

Biomarker	Clinical Use	Outcomes	Limitations	Reference
CTC	Predictive	Low baseline CTC correlated with a rapid response to immunotherapy ↓ CTCs in immunotherapy treated pts corelated with response to treatment and ↑ OS	Low detection sensitivity, particularly in early-stage melanoma	[115,117]
	Prognostic	≥1 CTC was independently associated with melanoma progression, relapse, OS, DMFS, and MSS		[116,117,118,119]
cfDNA	Prognostic	↑ baseline cfDNA was associated with the presence of metastases, ↑ AJCC stage, ↑ mortality risk, and ↓ OS	Low detection sensitivity	[129,130]
	Predictive	Alterations in cfDNA levels corresponded with treatment-induced changes in tumor size		[130]
ctDNA	Prognostic	Baseline ctDNA correlated with ↓ RFS and ↑ relapse risk postoperatively ctDNA was linked to worse DMFS	Often undetectable in early stages of melanoma	[144]
ctDNA	Predictive	Low baseline ctDNA correlated with better therapeutic response and ↑ PFS in pts treated with targeted therapy orimmunotherapyy No detectable ctDNA + complete response on imaging after halting immunotherapy due to toxicity correlated with long-term control of the disease ctDNA detection was associated with lower RFS and worse DMFS		[141,142,143,144]
cfRNA	Diagnostic Prognostic Predictive	↓ baseline cfRNA was linked to ↑ PFS and ↑ OS cfRNA during treatment was higher among NRs compared to RS, regardless of therapy type or mutation subtype	Difficult to detect [low cfRNA quantities, its tendency to degradation]	[147]
miRNAs	Diagnostic Prognostic	miRNA levels were significantly different in melanoma pts compared to controls and correlated with TNM stage	Validation studies are needed	[154,155,156]
	Predictive	miR-206 levels correlated with the response to treatment		[154]
Circulating immune cells	Predictive	The extent of T_ex_ cell reinvigoration relative to pretreatment tumor burden correlated with response to PD-1 therapy ↑ ALC, ↑ CD4+, and ↑ CD8+ T cells during ipilimumab treatment were linked to better survival outcomes ↑ baseline CD8 EM1 was linked to ↑ OS and better response rates to ipilimumab treatment ↑ levels of late-stage differentiated CD8 cells were associated with ↓ OS	Low abundance of certain immune cells and technical limitations	[161,162,163]

Abbreviations: ↓: decreased; ↑: increased; AJCC: American Joint Committee on Cancer; cfDNA: cell-free DNA; cfRNA: cell-free RNA; CTC: circulating tumor cells; ctDNA: circulating tumor DNA; DMFS: distant metastasis-free survival; miRNAs: microRNAs; MSS: melanoma-specific survival; NRs: non-responders; OS: overall survival; pts: patients; PFS: progression-free survival; RS: responders; Tex cells: exhausted-phenotype CD8 T cells; TNM: tumor/node/metastasis; ALC: absolute lymphocyte count; CD8 EM1: CD8 effector-memory type 1 T-cells.
